# *Hcn1* Is a Tremorgenic Genetic Component in a Rat Model of Essential Tremor

**DOI:** 10.1371/journal.pone.0123529

**Published:** 2015-05-13

**Authors:** Yukihiro Ohno, Saki Shimizu, Ayaka Tatara, Takuji Imaoku, Takahiro Ishii, Masashi Sasa, Tadao Serikawa, Takashi Kuramoto

**Affiliations:** 1 Laboratory of Pharmacology, Osaka University of Pharmaceutical Sciences, Takatsuki, 569–1094, Japan; 2 Department of Physiology and Neurobiology, Graduate School of Medicine, Kyoto University, Kyoto, 606–8501, Japan; 3 Nagisa Clinic, Hirakata, Osaka, 573–1183, Japan; 4 Institute of Laboratory Animals, Graduate School of Medicine, Kyoto University, Kyoto, 606–8501, Japan; National Institute of Genetics, JAPAN

## Abstract

Genetic factors are thought to play a major role in the etiology of essential tremor (ET); however, few genetic changes that induce ET have been identified to date. In the present study, to find genes responsible for the development of ET, we employed a rat model system consisting of a tremulous mutant strain, TRM/Kyo (TRM), and its substrain TRMR/Kyo (TRMR). The TRM rat is homozygous for the tremor (*tm*) mutation and shows spontaneous tremors resembling human ET. The TRMR rat also carries a homozygous *tm* mutation but shows no tremor, leading us to hypothesize that TRM rats carry one or more genes implicated in the development of ET in addition to the *tm* mutation. We used a positional cloning approach and found a missense mutation (c. 1061 C>T, p. A354V) in the hyperpolarization-activated cyclic nucleotide-gated 1 channel (*Hcn1*) gene. The A354V HCN1 failed to conduct hyperpolarization-activated currents *in vitro*, implicating it as a loss-of-function mutation. Blocking HCN1 channels with ZD7288 *in vivo* evoked kinetic tremors in nontremulous TRMR rats. We also found neuronal activation of the inferior olive (IO) in both ZD7288-treated TRMR and non-treated TRM rats and a reduced incidence of tremor in the IO-lesioned TRM rats, suggesting a critical role of the IO in tremorgenesis. A rat strain carrying the A354V mutation alone on a genetic background identical to that of the TRM rats showed no tremor. Together, these data indicate that body tremors emerge when the two mutant loci, *tm* and *Hcn1^A354V^*, are combined in a rat model of ET. In this model, HCN1 channels play an important role in the tremorgenesis of ET. We propose that oligogenic, most probably digenic, inheritance is responsible for the genetic heterogeneity of ET.

## Introduction

Essential tremor (ET) is one of the most common movement disorders. The major manifestation of ET is a postural and/or kinetic (action) tremor, which predominantly affects the hands, head, and vocal organs at a frequency of 4–12 Hz [1]. A recent meta-analysis using population-based studies estimated a pooled prevalence of ET (at all ages) of 0.9% [2]. Despite intensive research, few advances have been achieved in our understanding of the etiology of ET. Certain environmental factors may be at least partly involved in nonfamilial forms of ET [3]. However, a family history of ET and higher concordance rates in monozygotic than dizygotic twins support a major role for genetic factors in the development of ET [4].

Identification of causative genes for ET gives an insight into its pathogenesis and treatment. To date, several candidate genes have been identified. Linkage studies in families have revealed three ET-associated loci on chromosomes 3q13 (*ETM1*), 2p25-p22 (*ETM2*), and 6p23 (*ETM3*), but no gene with causative mutations has been reported [5,6,7]. A common variation in the dopamine D_3_ receptor gene (*DRD3*) within the *ETM1* locus has been suggested as a susceptibility factor for ET [8,9], but this association has not been replicated consistently across studies [10]. The common sequence variants in *LINGO1* and *SLC1A2* are also associated with ET [11,12], but the significance of these findings remains unclear [13,14].

Alternative approaches to the search for candidate genes for ET rely on research using animal models of the disorder. GABA_A_ receptor alpha subunit (*Gabra1*) knockout mice exhibit kinetic tremor and impaired motor coordination, resembling the symptoms of ET in human patients [15]. However, no change in the risk of ET has been found after analyzing GABA_A_ receptor genes in humans. To date, the *Gabra1* knockout mouse is the only available genetic animal model of ET, although more than 500 mutations are known to cause tremulous behavior in mice and rats (Mammalian Phenotype Browser, http://www.informatics.jax.org/searches/MP_form.shtml; Rat Genome Database, http://rgd.mcw.edu/). Therefore, characterization of such mice and rats as models of ET would benefit the search for candidate genes for the disorder.

TRM/Kyo rats, homozygous for the tremor (*tm*) mutation, were derived from a mutant showing body tremors and curled hair found in an outbred colony of Kyo:Wistar rats at the Institute of Laboratory Animals, Graduate School of Medicine, Kyoto University in 1980 [16]. The *tm* mutation was identified as a ~240-kb genomic deletion on rat chromosome 10, of which 13 genes have been mapped [17,18]. However, tremor resistant TRMR/Kyo rats, a substrain of TRM/Kyo, were found not to develop body tremors despite carrying the *tm* deletion. Therefore, our starting hypothesis was that TRM/Kyo rats carry at least one other gene that conveys vulnerability. TRMR/Kyo rats lack this susceptibility gene and thus show no tremor. We designated the *tm* deletion, one of the causative loci, as tremor 1 (*trm1*) and the other predicted locus as tremor 2 (*trm2*).

Here, we identify *trm2* as a missense mutation of the *Hcn1* gene. Electrophysiological and behavioral analyses reveal that HCN1 channels in the inferior olive (IO) play a crucial role in the pathogenesis of ET.

## Materials and Methods

### Rats and genomic DNA

TRM/Kyo (TRM), TRMR/Kyo (TRMR), and WTC/Kyo (WTC) rats, and the genomic DNA of 139 inbred rat strains to be used for mutation screening, were provided by the National BioResource Project—Rat (NBRP-Rat) (Kyoto, Japan). All animal experiments were approved by the Animal Research Committees of Kyoto University and Osaka University of Pharmaceutical Sciences, and were conducted according to the Committees’ regulations on animal experimentation. All surgery was performed under anesthesia, and all efforts were made to minimize suffering.

### Evaluation of tremor and effects of anti-tremor agents

TRM rats (5–7 weeks of age) were given the β receptor antagonist propranolol (30 mg/kg i.p.; Sigma-Aldrich, St. Louis, MO, USA), GABA_A_ receptor stimulant phenobarbital (Phenobal, 20 mg/kg i.p.; Daiichi Sankyo Co. Ltd., Tokyo, Japan) or muscarinic acetylcholine receptor antagonist trihexyphenidyl (3 mg/kg i.p.; Sigma-Aldrich) and placed individually in an observation box (25 × 42 × 20 cm). Tremor duration and intensity were estimated in each 1-min observation period, immediately before and 15, 30, 45 and 60 min after drug administration. The tremor intensity was evaluated using a four-point ranked scale (0, none; 1, weak; 2, moderate; 3, marked). Recording of electromyograph (EMG) activity was conducted under freely moving conditions, as described previously [19].

### Genetic mapping of *trm2*

Since they are homozygous for the *tm* deletion, the TRM and TRMR strains are both sterile [18]. Therefore, we used *tm*-heterozygous TRM and TRMR rats for the genetics experiment. [TRMR (*tm*/+) × TRM (*tm*/+)] F1 (*tm*/+) female rats were backcrossed to TRM (*tm*/+) rats to produce N2 progeny. Among them, 154 rats homozygous for the *tm* deletion were identified at 3 weeks of age by their curled whiskers and coat [16]. Rats homozygous for *trm2* were identified at 5 weeks of age, when body tremor was obvious. A previous study showed that 17 simple sequence length polymorphism markers in six genomic regions on chromosomes 2, 4, 5, 9, 12, and X were polymorphic between TRM and TRMR rats [20]. To finely map the *trm2* gene, three single nucleotide polymorphism (SNP) markers were used [21] (S1 Table). To find the mutation, reverse-transcription polymerase chain reaction (RT-PCR) and direct sequencing of the PCR products were carried out as described previously [22].

### Electrophysiology

Mouse wild-type and A354V-mutant *Hcn1* cDNA cloned in an oocyte expression vector (pBF) were transcribed *in vitro*. Electrophysiological studies using Xenopus oocytes were performed as described previously [23].

### Fos immunohistochemistry

TRM rats (5–6 weeks of age, n = 4) or WTC rats (5–6 weeks of age, n = 5) were used for immunochemical analysis of Fos protein expression. The number of Fos-immunoreactive cells was determined as described previously [24,25] in the following regions: 1) the cerebral cortices [motor cortex (MC), sensory cortex (SC), medial (MPtA) and lateral parietal association cortex (LPtA), granular insular cortex (GIC), piriform cortex (Pir), auditory cortex (AuC), and dorsolateral entorhinal cortex (DLEnt)]; 2) the limbic regions [CA1, CA2, CA3 and dentate gyrus (DG) of the hippocampus, basomedial amygdaloid nucleus (BMP)]; 3) the basal ganglia and brainstem regions [caudate-putamen (CPu), globus pallidus (GP), anteromedial (AM), centromedial (CM) and ventral posteromedial (VPM) thalamic nucleus, subthalamic nucleus (STh), anterior hypothalamic area (AHA), dorsomedial (DMH) and ventromedial (VMH) hypothalamic nucleus, medial tuberal nucleus (Mtu), parvocellular part of the red nucleus (RPC), substantia nigra pars reticulata (SNR), inferior olive (IO) and caudal spinal trigeminal nucleus (Sp5C)].

### Microinjection of an HCN1 channel antagonist

Microinjections were performed in rats as described previously [26]. TRMR rats (5–6 weeks of age, n = 15) or WTC rats (5–6 weeks of age, n = 8) were anesthetized with pentobarbital (50 mg/kg, i.p.) and fixed in a stereotaxic instrument (Narishige, SR-6, Tokyo, Japan). A stainless steel guide cannula was inserted into the lateral ventricle (LV; A: −1.7, L: 1.5, H: −3.5) [24]. After a recovery period, a selective HCN1 channel antagonist ZD7288 (4-ethylphenylamino-1,2-dimethyl-6-methylaminopyrimidinium chloride, 30 μg/20 μl/rat; Tocris, Bristol, UK) was injected into the LV under freely-moving conditions. The control animals were given the same volume of vehicle alone. Following behavioral observation, animals were sacrificed under anesthesia with pentobarbital (80 mg/kg, i.p.), and the brains were removed for subsequent Fos expression analysis.

### Electrical inferior olive (IO) lesion study

TRM rats (aged 6 weeks, n = 5) were anesthetized with pentobarbital (50 mg/kg, i.p.) and fixed in a stereotaxic frame. A bipolar concentric electrode was then inserted into the IO (A: −14.2, L: ± 1.0; H: −6.5) [24] and a direct current of 1 mA was delivered to the bilateral IO for 15 s.

### Statistical analysis

Data are expressed as the mean ± SEM. Statistical significance of differences among multiple groups was determined by one-way analysis of variance and Tukey’s post-hoc test (for parametric data) or Kruskal—Wallis test followed by the Steel—Dwass post-hoc test (for nonparametric data). Comparisons between two groups only were determined by Student’s *t*-test. A *P* value of less than 0.05 was considered statistically significant.

## Results

### TRM rats exhibit a phenotype similar to human ET

**Fig 1:**
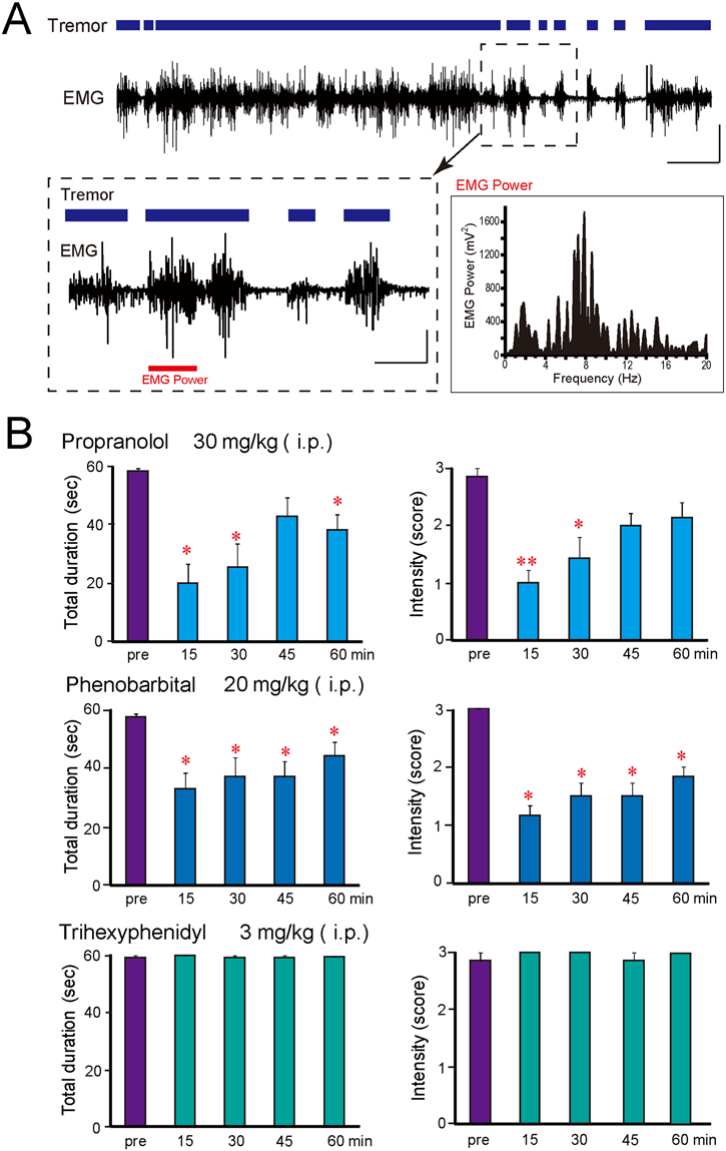
TRM rat as a model of essential tremor. A: Representative EMG from TRM rats. Tremor is shown as a bold line above EMG. Lower panels show magnified EMG and its power frequency analysis (red bar). Calibration: 100 μV and 20 s (upper panel), 50 μV and 5 s (lower panel). B: Effects of anti-tremor agents on tremor incidence in TRM rats. Data are presented as the mean ± SEM of seven (propranolol and trihexyphenidyl) or six (phenobarbital) animals. *P<0.05, **P<0.01, *vs*. pre-drug control levels (pre).

Tremors in TRM rats appeared during movement (kinetic tremor) and were especially prominent in the forelimbs, upper trunk, head, and neck (S1 Video). No gender differences were observed in the duration or intensity of tremor (data not shown). EMG recordings showed a synchronous association of EMG discharges with appearance of tremor. EMG power analysis revealed that tremor in TRM rats had a power peak frequency of 7–8 Hz (Fig 1A).

To evaluate the pharmacological responses of TRM rats to anti-tremor drugs, we administered drugs used for ET and for Parkinson’s disease tremor. Propranolol and phenobarbital significantly inhibited tremor duration and intensity (propranolol, intensity: *P* = 0.0013, duration: *P* = 0.0010; phenobarbital, intensity: *P* = 0.0008, duration: *P* = 0.0076); in contrast, the antiparkinsonian agent trihexyphenidyl failed to affect tremor (Fig 1B). Thus, TRM rats showed spontaneous and intensive tremor that resembles human ET symptomatically and pharmacologically, and were considered to be useful as an animal model of ET.

### *Trm2* is associated with a missense mutation in the *Hcn1* gene

TRM rats exhibit tremors, whereas rats of the substrain TRMR do not (S1 Video). To identify *trm2*, we employed a positional cloning approach. Among the 154 backcross progeny, 76 animals (32 male, 44 female) showed tremors and were identified as *trm2*/*trm2* homozygotes. The remaining 78 animals (39 male, 39 female) showed no tremor and were identified as *trm2*/+ heterozygotes. This indicated that *trm2* was a single autosomal recessive gene.

**Fig 2:**
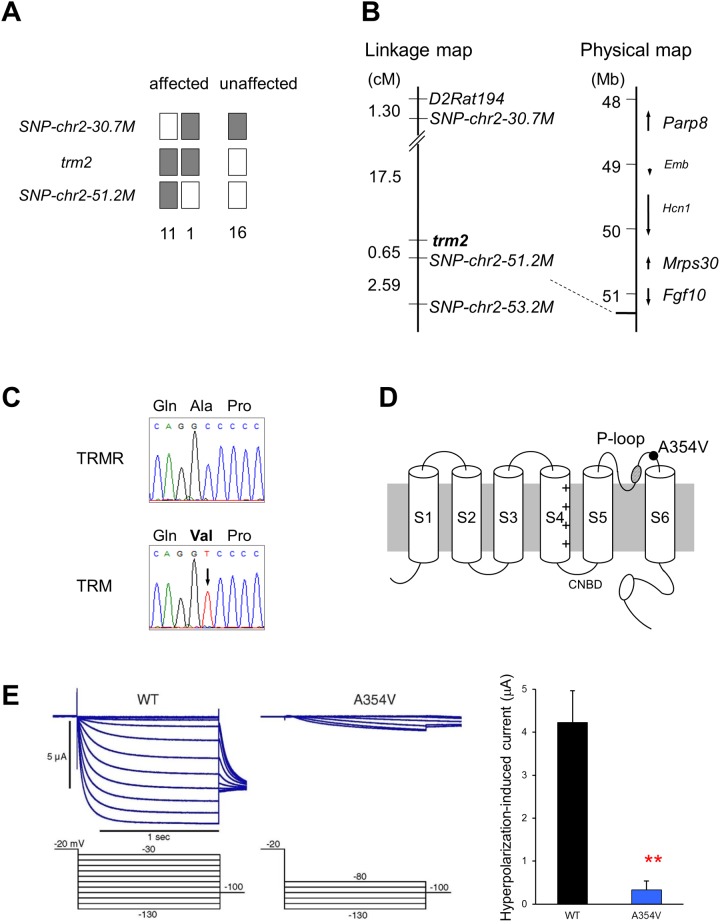
Identification of *trm2* mutation. A: Distribution of haplotypes around *trm2* in (TRM × TRMR)F1 × TRM backcross progeny. White boxes, animals heterozygous for TRM alleles. Black boxes, animals homozygous for TRM alleles. Number of backcross progeny specified underneath the haplotypes. B: Linkage and physical maps including *trm2*. *Parp8*, poly (ADP-ribose) polymerase family, member 8; *Emb*, embigin; *Mrps30*, mitochondrial ribosomal protein S30; *Fgf10*, fibroblast growth factor 10. C: Sequencing analysis in TRMR (upper) and TRM (lower) rats. A nucleotide change from C to T located in *Hcn1* exon 4 is indicated by arrow. The mutation results in an amino acid substitution of alanine (Ala) with valine (Val) at codon 354 of the HCN1 protein. D: Schematic representation of the HCN1 channel subunit. P-loop, pore region; CNBD, cyclic nucleotide-binding domain; black circle, A354V substitution located in the pore region. E: Representative current recordings of wild-type and A354V HCN1 channels. Right panel, hyperpolarization-induced currents measured at the end of the step pulse (-120 mV). Data are presented as the mean ± SEM of seven (wild-type) or six (A354V) experiments. **P<0.01 *vs*. wild-type.

The *trm2* locus was mapped 0.65 cM proximally to a SNP marker located at 51.2 Mb on chromosome 2 (Fig 2A). This was the narrowest region identifiable, because no markers showing polymorphism between TRM and TRMR rats were available within the *trm2* interval. We therefore estimated the size of *trm2* by extrapolating from genetic map units (cM). Our previous genetic studies indicated that 1 cM corresponded to 0.9–2.0 Mb [22,27]. Thus, to avoid overlooking candidate genes, we estimated *trm2* to be 2.6 Mb in size and span 48.6–51.2 Mb on chromosome 2. Within this interval, five characterized genes (poly ADP-ribose polymerase family member 8 (*Parp8*), embigin (*Emb*), *Hcn1*, mitochondrial ribosomal protein S30 (*Mrps30*), and fibroblast growth factor 10 (*Fgf10*)) and eight uncharacterized genes were mapped (Fig 2B). *Emb* promotes sprouting of motor nerve terminals at the neuromuscular junction [28] and *Hcn1* is expressed in the central nervous system and encodes an ion channel implicated in spontaneous rhythmic activity [29,30,31]. We therefore considered *Emb* and *Hcn1* to be good candidates, and sequenced their cDNA. Although no nonsynonymous nucleotide difference was found in *Emb* between the two strains, we observed a C-to-T nucleotide substitution at the 1,061st nucleotide of the *Hcn1* coding sequence (Fig 2C). This substitution was located in exon 4 of the *Hcn1* gene and resulted in an amino acid substitution of alanine (GCC) to valine (GTC) at residue 354 of the HCN1 protein. This Ala354Val (A354V) amino acid alteration was located within the pore segment between the fifth (S5) and sixth (S6) transmembrane domains, a region important for ion permeability [32] (Fig 2D).

To examine whether the A354V mutation was unique to TRM rats, genomic DNA from 139 inbred rat strains was screened. Of these, only the WTC and WKYO/Kyo strains carried the mutation. WTC is a coisogenic strain of TRM: their genomes are identical except at the *tm* deletion (*trm1*). WKYO rats were derived from the Kyo:Wistar colony. Neither the WTC nor WKYO strain show tremor behavior [33,34]. These findings suggested that the *Hcn1* ^*A354V*^ mutation occurred in the Kyo:Wistar stock and was specific to the TRM-related strains. Therefore, it is likely that the *Hcn1* ^*A354V*^ mutation causes ET in the TRM rats and, importantly, its effects on tremor development appear when it is combined with *trm1*.

### Electrophysiological properties of the A354V HCN1 channel

To examine the effects of the A354V mutation on HCN1 channel function, we performed electrophysiological analysis. Mouse wild-type and A354V HCN1 were expressed in Xenopus oocytes and currents evoked by the hyperpolarized step pulses (voltage: −30 to −130 mV; duration: 1200 ms) were recorded. Although wild-type HCN1 showed robust inward currents by hyperpolarizing voltage steps, A354V HCN1 showed almost no inward current. Currents induced by hyperpolarization (i.e. −120 mV) in the A354V HCN1 were significantly smaller than those in the wild-type HCN1 channel [A354V: 0.33 ± 0.21 μA (n = 6); wild-type: 4.23 ± 0.74 μA (n = 7); *P* = 0.0039] (Fig 2E). These findings suggested that the *Hcn1* ^*A354V*^ variant is a loss-of-function mutation in the rat *Hcn1* gene.

### Fos expression analysis in TRM rats

**Fig 3:**
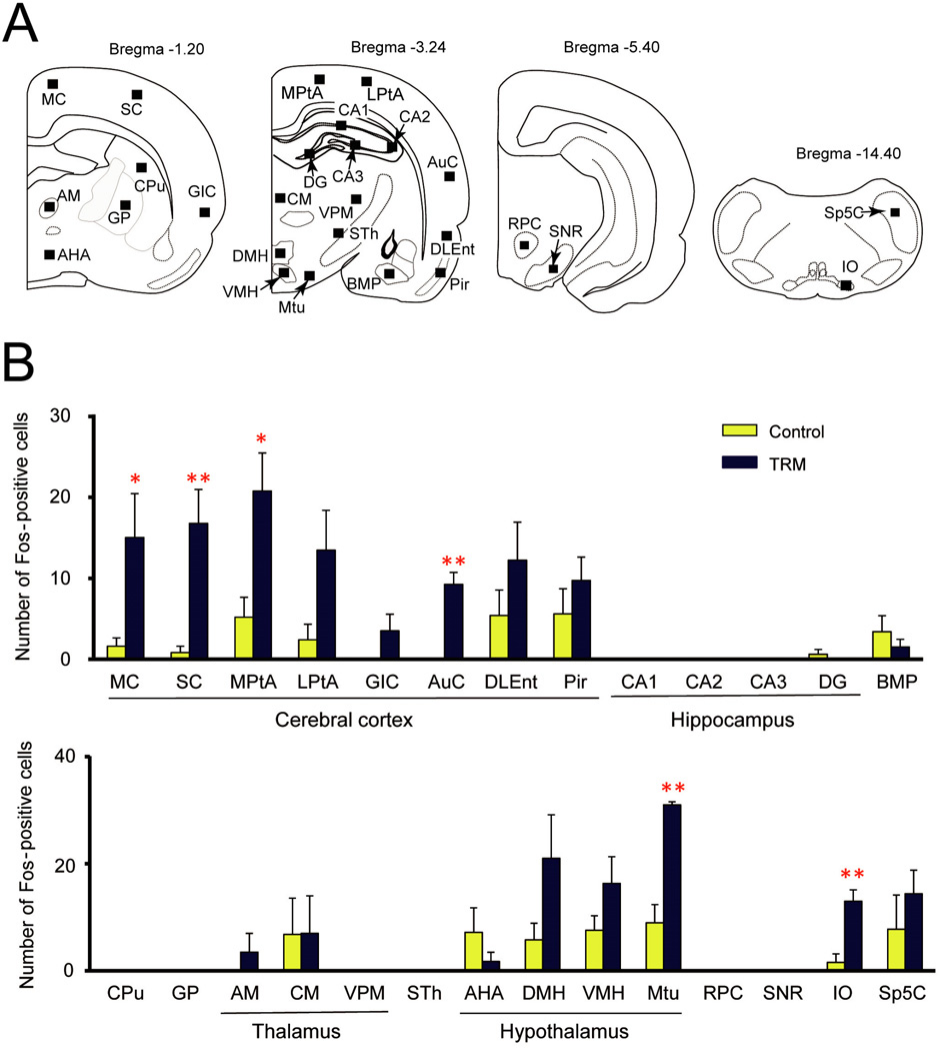
Immunohistochemical analysis of Fos expression in TRM rats. A: Schematic illustrations of brain sections selected for quantitative analysis of Fos-immunoreactivity. Anteroposterior distance from bregma is shown above each brain section. Filled boxes in each section indicate the areas analyzed. B: Numbers of Fos-immunoreactive neurons in various brain regions. Data show the mean ± SEM of four (TRM) or five (WTC) rats. *P<0.05, **P<0.01 *vs*. control (WTC).

To explore the brain regions related to tremor generation, we analyzed regional expression of Fos protein, a biological marker of neural excitation, in TRM rats (Fig 3A). A marked and significant elevation in Fos expression was observed in several regions of the cerebral cortex (MC: *P* = 0.0150; SC: *P* = 0.0041; MPtA: *P* = 0.0173; AuC: *P* = 0.0003), compared with nontremulous control (WTC) rats (Fig 3B). In the lower brain structures, Fos expression showed region-specific elevation in the Mtu (*P* = 0.0028) and IO (*P* = 0.0018) whereas no differences in Fos expression were observed in limbic regions, basal ganglia, thalamus or substantia nigra. These findings suggest that the generation of tremor in TRM rats is related to neural excitation in the IO, Mtu and/or some parts of the cerebral cortex.

### Blockade of HCN1 channels induces tremor in TRMR rats

**Fig 4:**
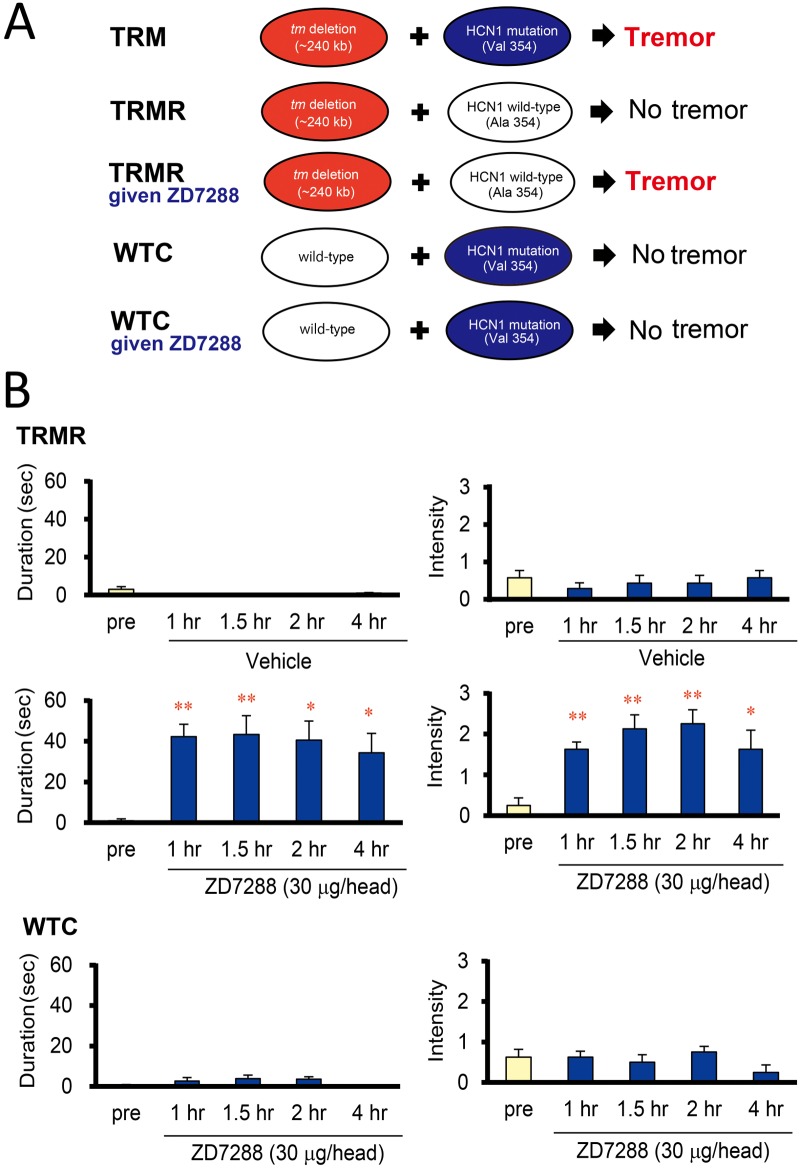
Tremor induction by the HCN1 channel blocker ZD7288 in TRMR rats. A: Genetic components responsible for tremor development in our rat model of ET. TRM rats, carrying both the *tm* deletion (red) and the *Hcn1* mutation (blue), showed body tremors. TRMR rats, carrying the *tm* deletion but not the *Hcn1* mutation, showed no body tremors, but body tremors were induced when the selective HCN1 channel blocker ZD7288 was administered (see B, this figure). WTC rats, carrying the *Hcn1* mutation but not the *tm* deletion, showed no body tremors with or without administration of ZD7288 (see B, this figure). B: Effects of ZD7288 on tremor induction in nontremulous TRMR rats. Duration and intensity of tremor observed in TRMR rats that received vehicle or ZD7288. Data are presented as the mean ± SEM of seven (vehicle) or eight (ZD7288) animals. *P<0.05, **P<0.01 *vs*. pre-treatment control levels (pre).

To determine whether HCN1 channel dysfunction is responsible for the generation of tremors *in vivo*, we evaluated the actions of a selective HCN1 channel blocker, ZD7288, in nontremulous TRMR and WTC rats, which carry the *tm* deletion or not, respectively, on identical genetic backgrounds (Fig 4A). When ZD7288 (30 μg/rat) was microinjected into the LV, intensive body tremors were evoked in all TRMR rats, but in none of the WTC rats (Fig 4B). The clinical manifestation of the induced tremor was comparable with that of the TRM rats and significant increases in the duration and intensity of the tremor persisted during the 4 h observation period (intensity: *P* = 0.0014; duration: *P* = 0.0025). Vehicle alone did not induce tremors in the TRMR rats. This result clearly demonstrates that *Hcn1* is the gene responsible for tremors (*trm2*) in our rat model of ET.

**Fig 5:**
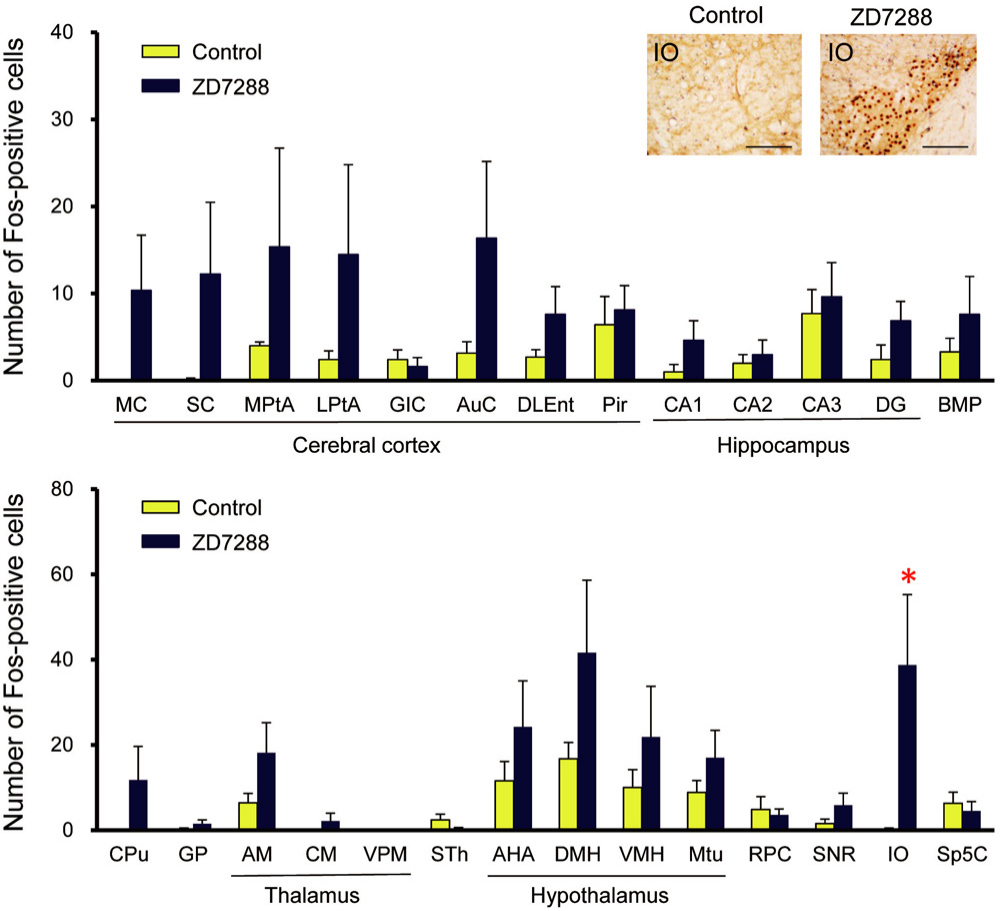
Immunohistochemical analysis of Fos expression induced by ZD7288 in TRMR rats. Effects of ZD7288 on regional Fos expression in TRMR rats. Inset: representative photographs of Fos-immunoreactive cells in the IO. Scale bar: 100 μm. Data are presented as the mean ± SEM of seven (vehicle) or eight (ZD7288) animals. *P<0.05 *vs*. control (vehicle).

To identify brain sites that were activated by ZD7288 in TRMR rats, we further analyzed regional Fos expression in ZD7288-treated TRMR rats. A marked and significant elevation in Fos expression was observed in the IO (*P* = 0.0495), compared with the TRMR rats given vehicle alone (Fig 5). These findings indicate that inhibition of HCN1 channels by ZD7288 causes excitation of IO neurons concomitantly with the generation of tremors.

### Electrical IO lesioning diminishes tremor in TRM rats

**Fig 6:**
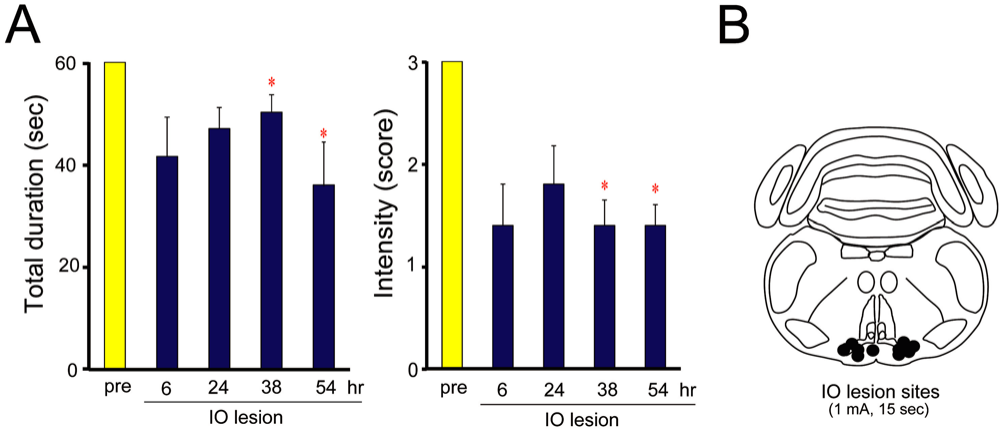
Tremor inhibition by IO lesioning in TRM rats. A: Effects of IO lesioning on tremor induction in TRM rats. The duration and intensity of tremor was significantly suppressed 38 and 54 h after IO lesioning. Data are presented as the mean ± SEM of five animals. *P<0.05 *vs*. pre-treatment control. B: Lesion sites in the brain sections.

To confirm whether the IO is responsible for the generation of tremors in TRM rats, we conducted an electrical lesion study of the IO. Electrical lesioning of the IO significantly inhibited the incidence of tremor in TRM rats (intensity: *P* = 0.0188; duration: *P* = 0.0270) (Fig 6). This inhibition persisted for more than 2 days following IO lesioning.

## Discussion

ET is a common movement disorder with a poorly understood etiology. In the present study, we established a rat model of ET and showed that body tremors emerge when two mutant loci (*Hcn1* ^*A354V*^ and the *tm* deletion) are present simultaneously. In addition, our results strongly support the notion that HCN1 channels in the IO play a crucial role in the pathogenesis of ET.

The tremor phenotype of TRM rats was similar to that of human ET. It was a spontaneous kinetic tremor with a frequency range (7–8 Hz) within that of ET (4–12 Hz). Tremors in TRM rats were selectively inhibited by drugs for ET but not parkinsonism. Thus, TRM rats were considered a good animal model of ET.

Using this model system, we have demonstrated that a combination of two genetic defects (the *tm* deletion and the *Hcn1* ^*A354V*^ mutation) is necessary for the generation of tremors, implying a digenic inheritance of ET tremorgenesis. Digenic inheritance is the simplest form of inheritance for genetically complex diseases [35]. Many human disease pedigrees show reduced penetrance when treated as monogenic in genetic analysis, but their inheritance could be explained more accurately by a two-locus model. Our present study clearly fits this model and suggests that some cases of ET in humans may be explained by digenic inheritance. Therefore, although familial ET shows high genetic heterogeneity [36], the digenic or oligogenic view of ET may provide new perspectives on ET genetics.

A missense mutation (A354V) in the *Hcn1* gene was associated with tremor expression in our rat model. HCN1 is one of the four isoforms of the HCN channel family (HCN1–4), which are expressed in the heart and brain and have different biophysical properties [37]. HCN subunits have six transmembrane domains, and functional channels consist of four subunits. The channels are permeable to sodium and potassium ions and are activated by membrane hyperpolarization. In the brain, they conduct *I*_*h*_ current, which contributes to spontaneous rhythmic activity and the stabilization of neuronal membrane potential against excitatory or inhibitory inputs [29,31]. Recently, *de novo* missense mutations in the *HCN1* gene were identified in patients with early infantile epileptic encephalopathy-24 [38]. Studies in rat models have also shown that *Hcn1* has a key role in epilepsy [39,40]. Our results provide the first evidence, to our knowledge, implicating HCN1 dysfunction in the pathogenesis of ET.

In our model, the IO is a brain region primarily involved in tremor generation. IO neurons exhibit synchronous oscillation activity and send such output signals to the cerebellum via climbing fibers [29]. This olivocerebellar pathway is thought to be involved in the control and coordination of movements, possibly including body tremor. Indeed, abnormal activation of the IO was shown by a functional imaging study in patients with ET [41]. In addition, activation of the IO and robust oscillatory output from it were observed in a harmaline-treated animal model of ET [29,42]. Furthermore, it is known that HCN1 channels are expressed densely in the IO [31]. When *I*_*h*_ currents are blocked, the IO neurons hyperpolarize and robust (abnormal) rhythmic oscillations are evoked [30]. It is therefore likely that a HCN1 dysfunction in the IO, which reduces or blocks *I*_*h*_ currents, may cause abnormal oscillations, and thereby induce ET in TRM rats.

The possible interaction of the *tm* deletion and *Hcn1* ^*A354V*^ mutation in the manifestation of tremor remains to be clarified. It is very likely that the *Hcn1* gene interacts with one or more genes within the *tm* deletion. The *tm* deletion spans nearly 240 kb and comprises at least 13 genes: sedoheptulokinase (*Shpk*), transient receptor potential vanilloid 1 and 3 (*Trpv1*/*3*), aspartoacylase (*Aspa*), spermatogenesis associated 22 (*Spata22*), seven olfactory receptor genes (*Olr1466*, *Olr1467*, *Olr1468*, *Olr1469*, *Olr1470*, *Olr1471*, *Olr1472)*, and one hypothetical gene (*LOC100359760*) [18]. SHPK is a carbohydrate kinase and functions as an immune modulator [43]. *Trpv1* and *Trpv3* encode transient receptor potential cation channels. TRPV1 responds to capsaicin, pH, and heat [44]. TRPV3 responds to camphor and warmth and is involved in hair follicle development [45,46]. *Spata* may play an important role in the regulation of meiotic phase I [18]. Olfactory receptors are known to be responsible for the recognition and G-protein-mediated transduction of odorant signals [47]. Mutant mice for *Trpv1*, *Trpv3*, and *Spata* genes are available, and show no apparent behavioral defects [44,45,48].

*Aspa* encodes aspartoacylase (EC 3.5.1.15), which hydrolyzes N-acetyl-L-aspartic acid (NAA) to aspartate and acetate. NAA is the second most concentrated molecule in the brain after glutamate. It has various functions in the brain, which include maintaining neuronal osmotic balance and serving as a source of acetate for lipid and myelin synthesis. It is also a precursor of N-acetylaspartylglutamate. In addition, NAA functions as an excitatory neurotransmitter by acting on metabotropic glutamate receptors [49,50]. *Aspa* knockout or mutant mice show behavioral defects such as tremor and abnormal myelination [51,52]. TRM rats also show abnormal myelination [53]. Myelin plays a critical role in the transmission of action potentials, and myelin-deficient mutant mice often show body tremors [54]. In addition, myelination is the primary factor in generating a uniform olivocerebellar conduction time [55]. Therefore, it is likely that *Hcn1* interacts with *Aspa* to produce ET in the TRM rat model.

We are currently working on producing *Aspa*- and *Hcn1*-knockout rats using genome editing technology such as transcription activator-like effector nucleases (TALENs). Examination of behaviors of *Aspa*/*Hcn1* double-knockout rats will confirm that the *Aspa* gene is essential for generating ET when it interacts with the *Hcn1*.

In summary, ET emerges in our rat model when two mutant loci (*trm1* and *trm2*) are combined. The IO plays an important role in this pathogenesis. We have identified *trm2* as *Hcn1*, which interacts with one or more genes within the *trm1* locus. The oligogenic, likely digenic, mode of inheritance observed in our rat model will be useful in human studies exploring ET in families, and may help to unravel the causative gene(s) for this condition.

## Supporting Information

S1 Table SNP markers used for fine mapping of *trm2*. (XLSX)

S1 Video Tremor phenotypes of TRM and TRMR rats. (MP4)
